# Supplementary material for: Is either direct photolysis or photocatalysed H-shift of peroxyl radicals a competitive pathway in the troposphere?
Source: R Soc Open Sci. 2020 Sep 9;7(9):200521. doi: 10.1098/rsos.200521 (PMC7540759; doi:10.1098/rsos.200521)
Supplement: Data for the photolysis cross section calculation [file rsos200521supp1.pdf]

SUPPLEMENTARY MATERIAL

Is Either Direct Photolysis or Photocatalyzed H-shift of Peroxyl Radicals A Competitive Pathways in the Troposphere?

Rashid R. Valiev[1,2], Theo Kurten[1,3]

1. University of Helsinki, Department of Chemistry, P.O. Box 55 (A.I. Virtanens plats 1), FIN-00014 University of Helsinki, Finland

2. Tomsk State University, 36, Lenin Avenue, 634050 Tomsk, Russia

3. University of Helsinki, Institute for Atmospheric and Earth System Research, Helsinki 00014, Finland

Contents

S1. Data for the photolysis cross section calculation.

S2. Data for the h-shift in D_0 state, h-shift and OH formation in D_1 state calculation.

S3. Cartesian coordinates of atoms in Å for reactant molecule and its transition state.

S1. Data for the photolysis cross section calculation

Table S1. d_e^{if} **is the electronic transition dipole moment of the strongest transition to a dissociative state,** R_0 **is the equlibribum bond length in the ground electronic state,** E_{if} **is the dissociation energy at the first dissociation limit,** A **and** B **are parameters fitted to the**

dissociative excited state with the largest transition dipole moment, m is reduced mass and ω is wavenumber corresponding to the last vibration along the breaking bond.

	d_e^{if} (a.u.)	R_0 (Å)	E_{if} (eV)	*A* (a.u)	*B* (a.u)	m (A.u.)	ω (cm^{-1})
$CH_3O\dot{O}$	0.66	1.3	2.7	3.74	1.39	10.6	1090
$HC(O)CH_2O\dot{O}$	1	1.3	2.7	3.74	1.39	12.6	928
$CH_3CH_2O\dot{O}$	0.67	1.3	2.7	3.74	1.39	11.8	959
$HC(O)O\dot{O}$	0.59	1.31	2.7	3.75	1.42	11.8	961
$CH_3C(O)O\dot{O}$	0.64	1.32	2.7	3.74	1.43	12.6	928

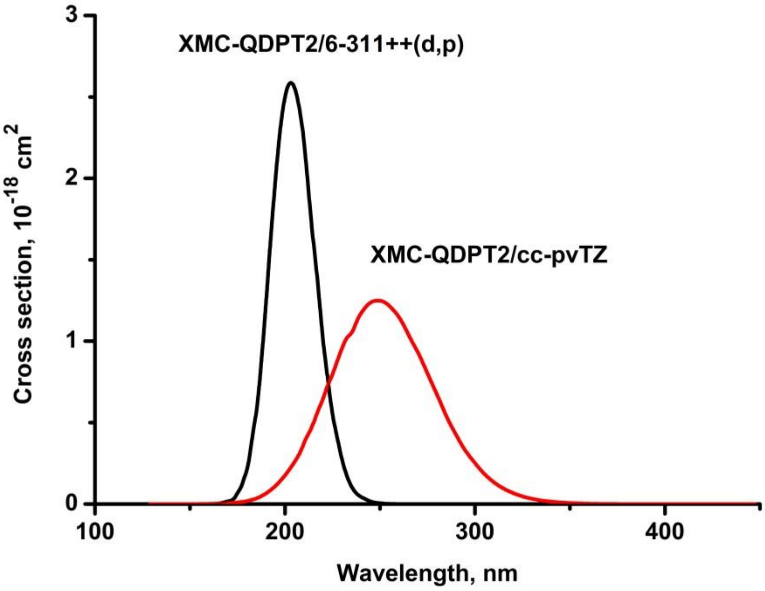

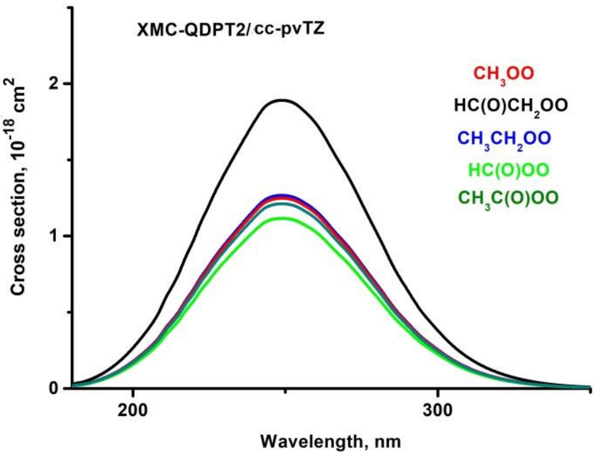

Fig. S1. The computed cross sections of photolysis at $CH_3O\dot{O}$.

S2. Data for the calculations on H-shifts in the D_0 state, and OH loss or H-shifts in the D_1 state.

Table S2.1. Activations barrier (*E_0*) for H-shifts in the D_0 state, activation barriers (*E_1*) in for H-shift or OH loss reactions the D_1 state, imaginary frequency (ω_0) and reduced mass (*m_0*) in the D_0 state, imaginary frequency (ω_1) and reduced mass (*m_1*) in D_1 state, Eckart correction factor (*κ_0*) in the D_0 state, Eckart correction factor (*κ_1*) in the D_1 state, and the rate constant of the H-shift in the D_0 state, $k_{H\text{-}shift}$, all computed at the XMCQPDT2/CASSCF(11,7)/6-311++G(d,p) level of theory.

	E_0 (kcal/mol)	E_1 (kcal/mol)	ω_0 (cm^{-1}) and m_0	ω_1 (cm^{-1}) and m_1	κ_0	κ_1	$k_{H\text{-}shift}$ (s^{-1})
$CH_3O\dot{O}$	48	35*	2054, 1.1	1146, 4.1	$9\cdot10^{5}$	5.8	$1.0\cdot10^{-16}$
$HC(O)CH_2O\dot{O}$	25	33	2802, 1.1	1981, 2.5	$1.1\cdot10^{7}$	$3.0\cdot10^{4}$	4.8
$CH_3CH_2O\dot{O}$	42	30	2800, 1.1	1910, 1.1	$1.1\cdot10^{11}$	$7.8\cdot10^{3}$	$8.5\cdot10^{-4}$
$HC(O)O\dot{O}$	42	42**	2110, 1.1	1133, 1.3	$1.1\cdot10^{6}$	5.5	$1.0\cdot10^{-12}$
$CH_3C(O)O\dot{O}$	35.6	28	2353, 1.1	1047, 2.9	$1.1\cdot10^{7}$	5.7	$5.0\cdot10^{-8}$

*Activation barriers and other data for the D_1 state correspond to the OH loss reaction, as a transition state for the 1,3 H-shift could not be found in this state for CH_3OO.

**Activation barriers and other data for the D_1 state correspond to the HO_2 loss reaction, as a transition state for the 1,3 H-shift could not be found in this state for $HC(O)O\dot{O}$.

Table S2.2. The activation barrier (*E_0*) in the D_0 state, activation barrier (*E_1*) in the D_1 state, imaginary frequency (ω_0) and reduced mass (*m_0*) in D_0 state, imaginary frequency (ω_1) reduced mass (*m_1*) in D_1 state, computed at XMCQPDT2/CASSCF(11,7)/6-311++G(d,p) and at XMCQPDT2/CASSCF(11,7)/cc-pvTZ level of theory for $CH_3O\dot{O}$.

	E_0 (kcal/mol)	E_1 (kcal/mol)	ω_0 (cm^{-1}) and m_0	ω_1 (cm^{-1}) and m_1
**6-311++G(d,p)**	48	35	2054, 1.1	1146, 4.1
**cc-pvTZ**	47	34	2155, 1.1	1643, 1.5

S3. Cartesian coordinates of atoms in Å for reactant molecule and transition state

1. D_0 state

1.1. $CH_3O\dot{O}$

RC

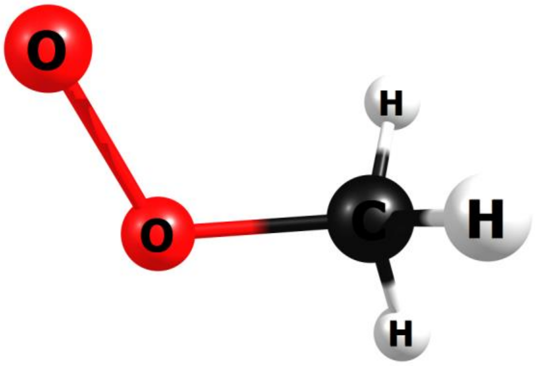

O	-0.002677551	0.559911422	0.328918530
O	-1.063994811	0.419442733	-0.463472202
C	0.941203967	-0.500749997	0.071759500
H	0.457687702	-1.459877309	0.255082600
H	1.762752712	-0.338245616	0.768263184
H	1.283306944	-0.432241771	-0.960826754

TS

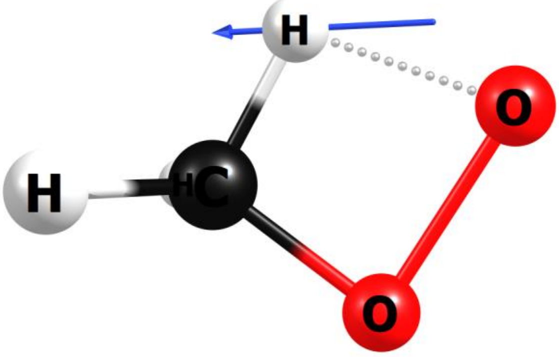

O	-0.002677551	0.628380861	0.068186899
O	-0.996925440	-0.554369017	0.121763838
C	0.995828602	-0.313712407	0.012861199
H	0.114300895	-1.235262885	0.056000481
H	1.593375879	-0.411230187	0.918560785
H	1.502270882	-0.396190282	-0.949869251

1.2. $HC(O)CH_2O\dot{O}$

RC

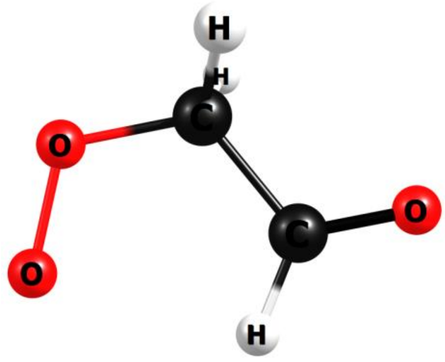

O	1.559390647	0.372554965	-0.026951945
O	1.823016194	-0.933754877	-0.013854552
C	0.137982290	0.679717803	-0.023760234
H	-0.033212914	1.305362218	0.856215901
H	-0.043710708	1.277802670	-0.920688756
C	-0.847707453	-0.481215899	-0.000547708
O	-2.037089748	-0.228460073	0.005152663
H	-0.456980063	-1.507315812	0.009303672

TS

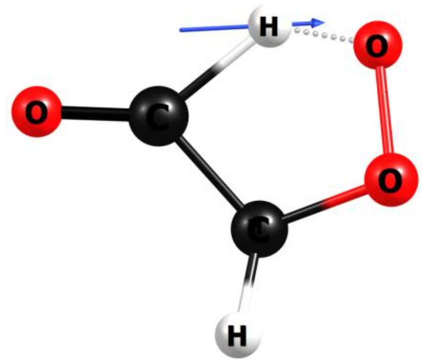

O	1.286349711	0.421756799	-0.345037978
O	1.334333149	-0.937738167	0.160586635
C	0.122036636	0.947766985	0.220589957
H	0.183632441	1.068766540	1.310029660
H	-0.134268274	1.883030342	-0.286355345
C	-0.894138130	-0.215961328	-0.016159758
O	-2.065409917	-0.261922365	-0.089938135
H	0.216457588	-1.105756568	0.014799610

1.3. $CH_3CH_2O\dot{O}$

RC

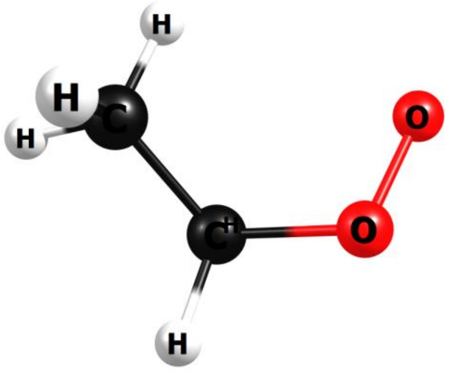

O	0.933543476	0.441964531	-0.317630583
O	1.597917633	-0.579732584	0.223666956
C	-0.382828576	0.556807221	0.289693542
H	-0.241274000	0.594215539	1.371540214
H	-0.745286773	1.520285868	-0.073730405
C	-1.276166327	-0.589450591	-0.137287145
H	-1.349238579	-0.629641335	-1.227097297
H	-0.880044044	-1.537721076	0.229480349
H	-2.279179057	-0.443878172	0.274194429

TS

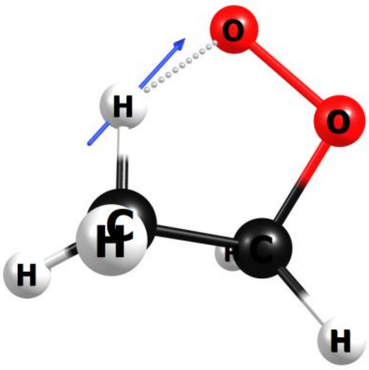

O	0.784451329	0.605358382	-0.298951374
O	1.133813444	-0.715182602	0.161898084
C	-0.549869984	0.761176145	0.218360004
H	-0.535827028	0.923337082	1.299445705
H	-0.960929639	1.634806425	-0.292750427
C	-1.192401603	-0.569222878	-0.116692900
H	-1.418105574	-0.722618762	-1.172539469
H	-0.046094654	-1.123240386	0.092281271
H	-1.944443908	-0.958807474	0.565525466

1.4. HC(O)OȮ

RC

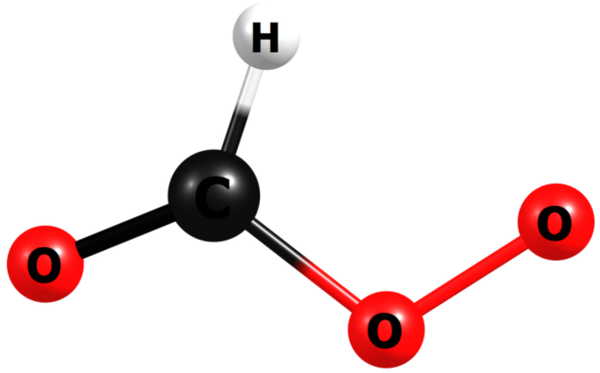

O	0.614271597	0.564444796	-0.000046038
C	-0.299883159	-0.520100801	0.000042334
O	-1.464527671	-0.315715084	-0.000342378
O	1.840118631	0.111992956	0.000383124
H	0.251568747	-1.461412933	-0.000016404

TS

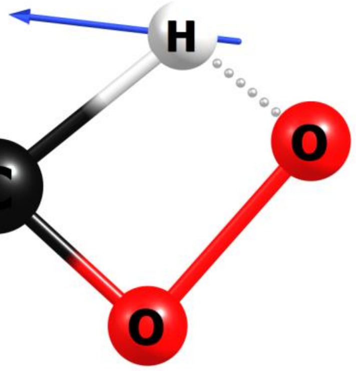

O	0.469684000	0.727731000	-0.000061000
C	-0.492852000	-0.163031000	0.000008000
O	-1.680859000	-0.115064000	0.000005000
O	1.528494000	-0.351564000	0.000039000
H	0.418558000	-1.110638000	0.000094000

1.5. $CH_3C(O)O\dot{O}$

RC

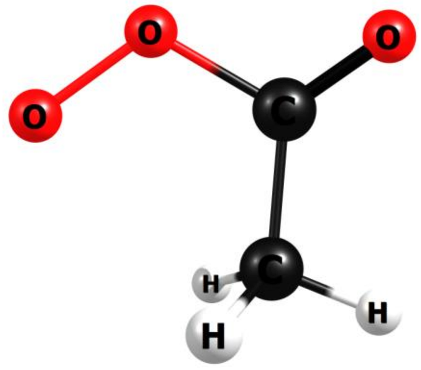

O	0.711969103	1.202660830	-0.890714102
C	-0.502952942	1.037925795	-0.109089454

O	-0.870926074	-0.089890478	0.005117795
C	-1.110531481	2.295750627	0.427452358
H	-0.409395890	2.795822619	1.098915565
H	-2.018183931	2.018318391	0.962138289
O	1.121188792	2.473538284	-0.949998263
H	-1.334772882	2.984471404	-0.389971087

TS

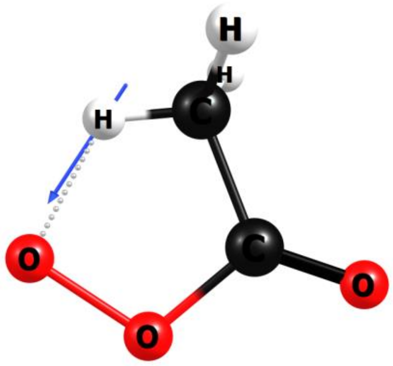

O	0.711969051	1.279678359	-1.058467574
C	-0.127715275	0.957017985	0.001539743
O	-0.246791545	-0.165495818	0.387914661
C	-0.810980732	2.196243382	0.496557106
H	-0.570958440	2.465712264	1.524715111
H	-1.880276643	2.210064927	0.290586694
O	0.643128002	2.737133307	-1.222551606
H	-0.239207018	2.940814888	-0.275942670

1. D_1 state

1.1. $CH_3O\dot{O}$

RC

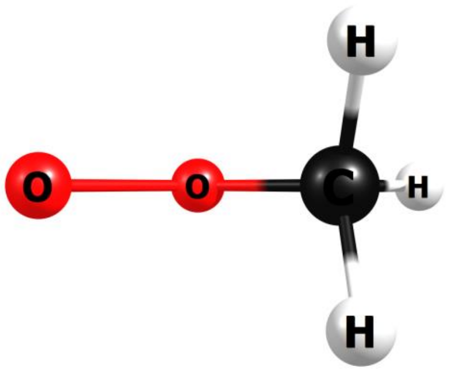

O	-0.002677551	0.574130387	0.361837849
O	-1.086618126	0.373075206	-0.517012866

C	0.914028184	-0.490343130	0.076279360
H	0.450719071	-1.458694996	0.281475616
H	1.745438883	-0.317061603	0.758055969
H	1.251768627	-0.432855497	-0.960906897

TS

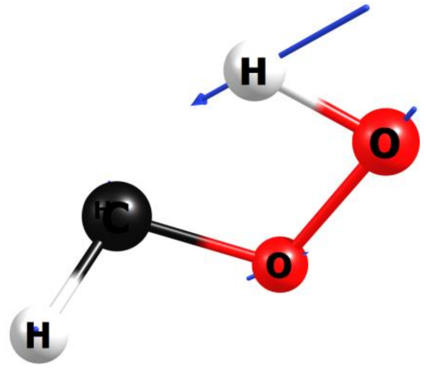

O	-0.002677551	0.483794308	0.015930118
O	-1.246911933	-0.287749861	0.086431664
C	0.999735251	-0.537283901	0.003233442
H	-0.673602184	-1.170120954	0.071597475
H	1.606235378	-0.321341039	0.890590059
H	1.563216666	-0.388050283	-0.928232921

1.2. HC(O)CH_2OȮ

RC

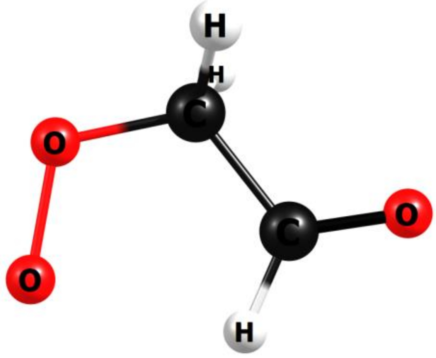

O	1.551878638	0.405488172	-0.027927570
O	1.782357607	-0.983943490	-0.012472563
C	0.140177869	0.721933811	-0.023952671
H	-0.055560838	1.333828957	0.860714325
H	-0.066867791	1.307209438	-0.923994178
C	-0.818187964	-0.460495009	-0.001231405
O	-2.014121058	-0.263921822	0.005413051
H	-0.389128517	-1.477908895	0.007960800

TS

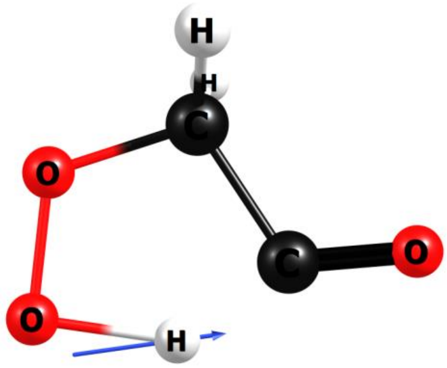

O	1.355829819	0.491477830	-0.026621639
O	1.476311436	-1.029311132	-0.009913560
C	0.020190742	0.952971552	-0.026964063
H	-0.102420620	1.560028922	0.876103327
H	-0.112170789	1.534117266	-0.945752501
C	-0.955278514	-0.324478801	0.000018036
O	-2.147764783	-0.168107887	0.005721167
H	0.369700003	-1.161059872	-0.002610223

1.3. $CH_3CH_2O\dot{O}$

RC

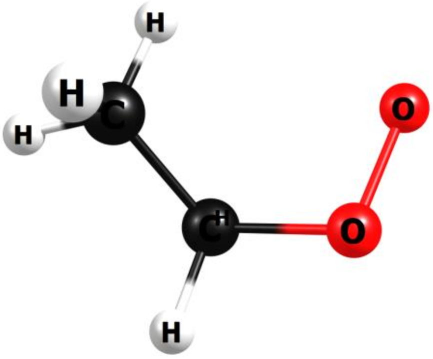

O	0.935604147	0.500704873	-0.318533180
O	1.581284209	-0.627577911	0.221993962
C	-0.377518304	0.558483660	0.283636757
H	-0.262348022	0.624274291	1.370323710
H	-0.758775336	1.509156158	-0.091970714
C	-1.262365422	-0.602889633	-0.128591543
H	-1.298592680	-0.679271734	-1.217715340
H	-0.894779227	-1.547427561	0.276550091
H	-2.276800593	-0.436326774	0.246656092

TS

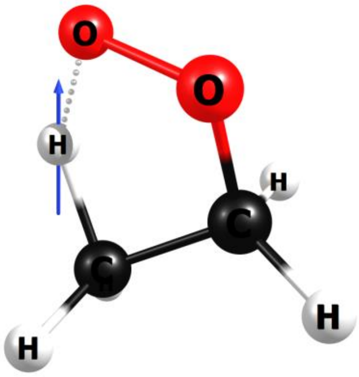

O	0.832864904	0.660282648	-0.047204860
O	1.134071537	-0.782157284	-0.013394609
C	-0.605047278	0.804475266	0.041140210
H	-0.836497991	1.315372973	0.979375118
H	-0.926063849	1.414803966	-0.807719129
C	-1.177115409	-0.595140950	-0.008861678
H	-1.571071496	-0.922547302	-0.968110186
H	-0.081557720	-1.166379067	0.064846472
H	-1.787687728	-0.903478074	0.836390828

1.4. HC(O)OȮ

RC

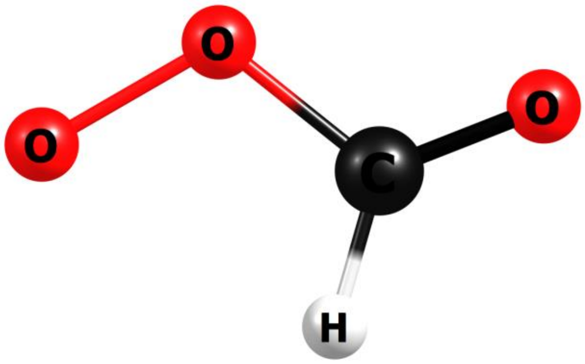

O	-0.692384500	-0.373302673	-0.393404048
O	-1.440983475	0.593283298	0.288450742
C	0.368036108	-0.863316138	0.349566525
H	0.424209099	-0.407725065	1.344167099
O	1.095149401	-1.687740209	-0.110271794TS

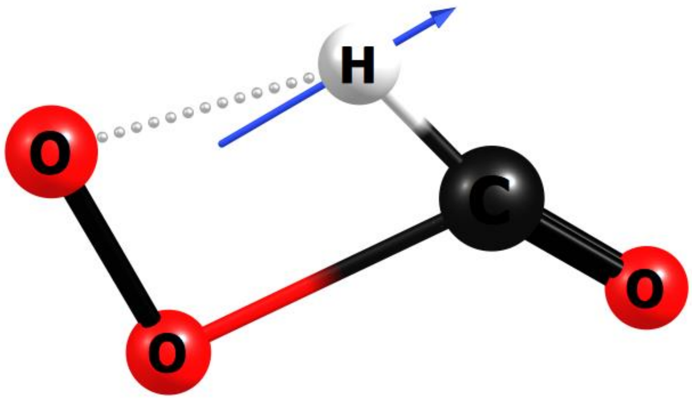

O	0.557743040	0.885904207	-0.495811826
C	-0.809363871	-0.570757910	0.212182455
O	-1.918783634	-0.347287245	0.288187059
O	1.519333936	-0.052583464	-0.307909222
H	0.293708475	-0.879641589	0.166641112

1.5. $CH_3C(O)O\dot{O}$

RC

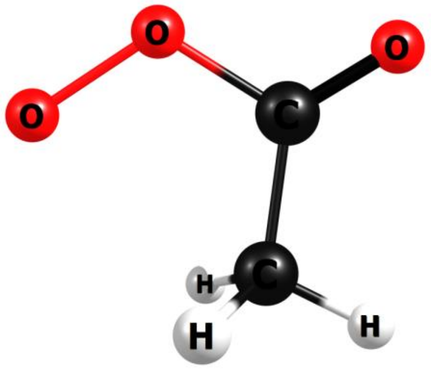

O	0.711969103	1.205728542	-0.843254321
C	-0.489898109	1.020476287	-0.110855423
O	-0.880858656	-0.100656979	0.001147870
C	-1.096243167	2.283180892	0.420580502
H	-0.382134938	2.778343122	1.082974206
H	-2.010439532	2.034397081	0.958141574
O	1.121986027	2.538977576	-0.942303992
H	-1.311363974	2.958143328	-0.412588770

TS

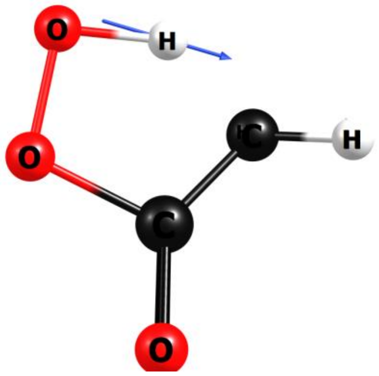

O	-0.531533135	-0.900733729	-0.212448980
C	0.633044793	0.060775020	0.013447009
O	1.657665326	-0.506639262	0.223685701
C	0.127253391	1.413830702	0.066966878
H	0.017644153	1.909367015	-0.898010764
H	0.570974608	2.019119630	0.852212991
O	-1.706355781	-0.164219966	-0.120243837
H	-1.189447386	0.845184927	0.136013008
